# Isolated posterior malleolus fracture: a rare injury mechanism

**DOI:** 10.11604/pamj.2015.20.123.6046

**Published:** 2015-02-12

**Authors:** Sancar Serbest, Uğur Tiftikçi, Haci Bayram Tosun, Engin Kesgin, Metin Karataş

**Affiliations:** 1 Department of Orthopaedics and Traumatology, Faculty of Medicine, Kırıkkale University, Kırıkkale, Turkey; 2 Department of Orthopaedics and Traumatology, Faculty of Medicine, Adiyaman University, Adiyaman, Turkey; 3 Anamed Private Hospital, Department of Orthopaedics and Traumatology, Mersin, Turkey; 4 Beypazari State Hospital, Department of Orthopaedics and Traumatology, Ankara, Turkey

**Keywords:** Ankle, fracture, posterior malleolar fractures, Ottawa Ankle Rules, Ankle sprain, isolated

## Abstract

Sprain of the ankle is undoubtedly a common injury during athletic activity, and the sprain can be also associated with fracture of the ankle. Isolated posterior malleolus fracture is a very rare condition, which is usually missed. Here, we are presenting a 37 years old female patient, who suffered injury secondary pressing on brake pedal during collision in a traffic accident. Clinical evaluation is based on Ottawa Ankle Rules and a fracture is diagnosed; patient is started on daily activities at postoperative Week 8. This study aims to emphasize that Ottawa Ankle Rules are usually efficient for evaluating fractures of ankle, but clinicians should always make a detailed physical examination.

## Introduction

Ankle is most commonly injured during sportive activities [1]. Among all patients referring to Emergency Room at U.S., sprain is the most common injury of ankle, while prevalence of ankle fracture is 0.1 to 0.2% [2]. Isolated posterior malleolus fracture is a rare form of ankle fractures, and plantar flexion and axial sprain is the underlying mechanism, which is not included in the classification systems [3]. Exact pathophysiological mechanism is unclear, although it is usually with ligament injury [4].

## Patient and observation

**Figure 1:**
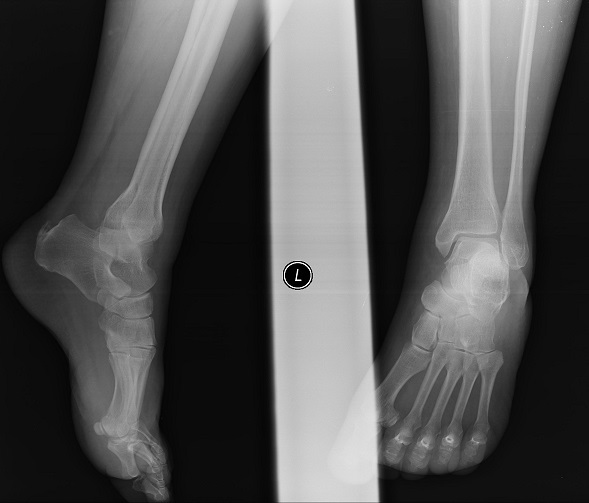
On direct x-ray examination in Emergency room. Anteroposterior (a) and lateral X-ray of the ankle

**Figure 2:**
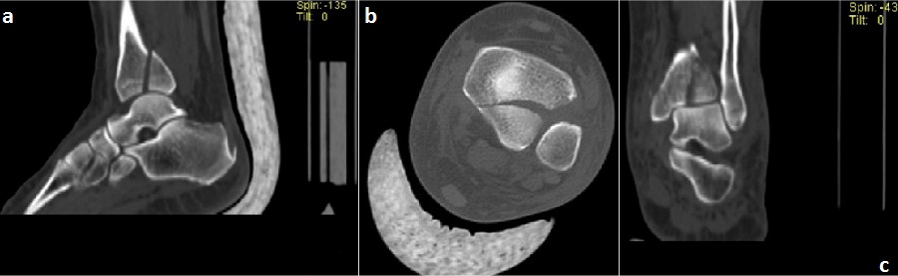
A bone fragment with 25% separation was observed on the posterior malleolus and the fragment was also extending to articular surface a) sagitta;l b) aksiyal; c) Koronal

**Figure 3:**
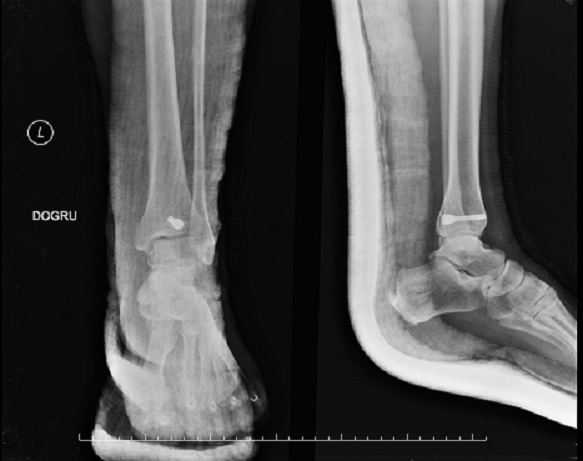
Closed reduction and One compression screw was fasted at posteroanterior orientation and the fracture was fixed. Anteroposterior (a) and lateral X-ray of the ankle

**Figure 4:**
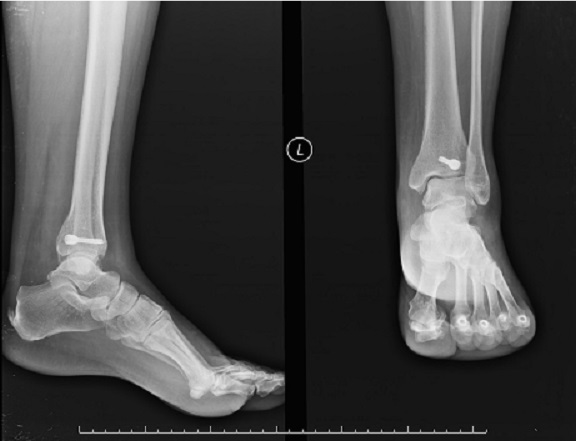
On final radiographies, union was verified

Wearing heals, the 37 years old female patient referred to Emergency room for pain in the right ankle secondary to pushing the brake pedal during a traffic accident. On the physical examination, patient was ambulatory, although minimal limping was observed in right lower limb, and no swelling was noted on the ankle. Local tenderness was not found at medial and lateral malleolar localizations. Fibular compression test was negative and no tenderness was palpated on the fibula. On baseline radiographies, no fracture was observed and soft tissue injury was considered (Figure 1). Patient referred to Orthopedics outpatient clinic 2 days later, since pain persisted at the ankle. Patient was examined again according to Ottawa criteria. In addition to findings identified above, patient could not walk even for 4 steps. Radiographies were re-evaluated. Computed tomography was scanned. A bone fragment with 25% separation was observed on the posterior malleolus and the fragment was also extending to articular surface (Figure 2). Spinal anesthesia was induced. One compression screw was fasted at posteroanterior orientation and the fracture was fixed (Figure 3). Ankle exercises were started at early postoperative course. Patient was mobilized with two crutches for 3 weeks without bearing load. Patient was allowed bearing load at Week 6, as long as patient could tolerate. Patient could be mobilized without crutches at Week 8. On final radiographies, union was verified and ROM of the ankle was intact (Figure 4). AOFAS (American Orthopedic Foot and Ankle Society) score was 96.

## Discussion

Isolated posterior malleolus fracture is a very rare condition, which is usually missed. It is first introduced by Tobin [5] in 1943 as “parachute jumper's fracture”. All fractures of ankle are associated by posterior malleolus fracture by 14 to 44 percent [6], while isolated posterior malleolus fractures account only 1% of them [7, 8]. Isolated posterior malleolus fractures are secondary to compression and axial loading mechanism, along with over-tension of the posterior syndesmosis ligament, and this type of fracture is not included in the classification system introduced by Lauge-Hansen [7]. Ebrahim et al. [9] demonstrated that symptomatic non-union of posterior malleolus fracture cannot be visualized on conventional anteroposterior radiographies and Lateral projection should be scanned while ankle is externally rotated by 50 degrees. Büchler et al. [10] conducted a study, where posterior malleolus fractures were evaluated on computed tomography (CT) scans and plain radiography, and authors demonstrated that CT has better reliability and accuracy. Conventional anteroposterior and lateral radiographies were scanned for our patient, who referred to Emergency Room, and posterior malleolus fracture was missed. Management of intra-articular fractures of the lower limb is to gain a functioning as close as possible to the native ROMs of the ankle. Fracture is reduced in a stable manner and patient should be mobilized and weight should be born as soon as possible in the postoperative course in order to achieve this aim. Reduction of posterior malleolus is very important, since the structure carries heavy load during walk, although it has a small intra-articular surface [11–14].

Although management of ankle fractures, which are associated with posterior malleolus fracture, has been defined by many authors, there is still no consensus on classification of the fracture, indications of surgery, operation technique and the best treatment algorithm, also including functional outcomes [15]. The widely accepted view is that the size of the fragment is the principal factor for the indication of surgery. If the fragment involves more than 25 to 30% of the articular surface, many surgeons recommend fixation for posterior malleolus fracture [13, 16–18]. There are also surgeons claiming that all posterior malleolus fractures should be fixed, since this approach will lead to better posterior syndesmotic stability. Heim [19] recommended surgical fixation for all posterior malleolus fractures, excluding tibial posterior rim fractures. Langenhuijsen et al. [20] recommended that all displaced posterior malleolus fractures should be fixed irrespective of the size, after lateral and medial malleolus are fixed. Donken et al. conducted a study with 20 years of follow-up period, and authors demonstrated that there is statistically significant relation between the severity of the displacement between tibiothalar contact area percent and fragments and clinical outcomes of isolated posterior malleolus fracture and conservative treatment is also associated with good clinical and radiological outcomes [4]. Atraumatic arthritis developed more frequently in fractures of the ankle, if they are associated with the posterior malleolus fracture. Stress-induced changes secondary to loading on articular surface are blamed for articular changes, which occur after posterior malleolus fractures [21]. Unnecessary examinations will not be ordered at emergency rooms and risk of missing the fracture will be minimized, if clinical evaluation is bade don Ottawa Ankle rules, which are introduced by Stiell et al. [22]. The fracture was also missed for the patient, who referred to emergency room and was diagnosed with soft tissue injury. Next, patient referred to Orthopedics outpatient clinic and fracture was considered and CT was ordered, when the patient was examined in the light of Ottawa Ankle Rules. Posterior malleolus fracture was visualized, which was involving >25% of the articular surface. Fixation was made using compression screw, which was placed on posteroanterior orientation.

## Conclusion

Recently, normal anatomic mortis and restoration of anatomic tibiothalar contact area are significant factors to gain a good functional outcome, after ankle injury is managed. Clinicians should always consider possibility of posterior malleolus fracture for patients, who refer to emergency room for pain and swelling during loading along with sprain of the ankle, although this type of fracture cannot be clearly visualized on radiographies.
